# Deep phenotyping: symptom annotation made simple with SAMS

**DOI:** 10.1093/nar/gkac329

**Published:** 2022-05-07

**Authors:** Robin Steinhaus, Sebastian Proft, Evelyn Seelow, Tobias Schalau, Peter N Robinson, Dominik Seelow

**Affiliations:** Exploratory Diagnostic Sciences, Berliner Institut für Gesundheitsforschung, Berlin 10117, Germany; Institut für Medizinische Genetik und Humangenetik, Charité – Universitätsmedizin Berlin, corporate member of Freie Universität Berlin and Humboldt-Universität zu Berlin, Berlin 13353, Germany; Exploratory Diagnostic Sciences, Berliner Institut für Gesundheitsforschung, Berlin 10117, Germany; Institut für Medizinische Genetik und Humangenetik, Charité – Universitätsmedizin Berlin, corporate member of Freie Universität Berlin and Humboldt-Universität zu Berlin, Berlin 13353, Germany; Medizinische Klinik mit Schwerpunkt Nephrologie und Internistische Intensivmedizin, Charité – Universitätsmedizin Berlin, corporate member of Freie Universität Berlin and Humboldt-Universität zu Berlin, Berlin 10117, Germany; Exploratory Diagnostic Sciences, Berliner Institut für Gesundheitsforschung, Berlin 10117, Germany; FB Mathematik und Informatik, Freie Universität Berlin, Berlin 14195, Germany; The Jackson Laboratory for Genomic Medicine, Farmington, CT 06030, USA; Institute for Systems Genomics, University of Connecticut, Farmington, CT 06030, USA; Exploratory Diagnostic Sciences, Berliner Institut für Gesundheitsforschung, Berlin 10117, Germany; Institut für Medizinische Genetik und Humangenetik, Charité – Universitätsmedizin Berlin, corporate member of Freie Universität Berlin and Humboldt-Universität zu Berlin, Berlin 13353, Germany

## Abstract

Precision medicine needs precise phenotypes. The Human Phenotype Ontology (HPO) uses clinical signs instead of diagnoses and has become the standard annotation for patients’ phenotypes when describing single gene disorders. Use of the HPO beyond human genetics is however still limited. With SAMS (Symptom Annotation Made Simple), we want to bring sign-based phenotyping to routine clinical care, to hospital patients as well as to outpatients. Our web-based application provides access to three widely used annotation systems: HPO, OMIM, Orphanet. Whilst data can be stored in our database, phenotypes can also be imported and exported as Global Alliance for Genomics and Health (GA4GH) Phenopackets without using the database. The web interface can easily be integrated into local databases, e.g. clinical information systems. SAMS offers users to share their data with others, empowering patients to record their own signs and symptoms (or those of their children) and thus provide their doctors with additional information. We think that our approach will lead to better characterised patients which is not only helpful for finding disease mutations but also to better understand the pathophysiology of diseases and to recruit patients for studies and clinical trials. SAMS is freely available at https://www.genecascade.org/SAMS/.

## Graphical Abstract

**Graphical Abstract:**
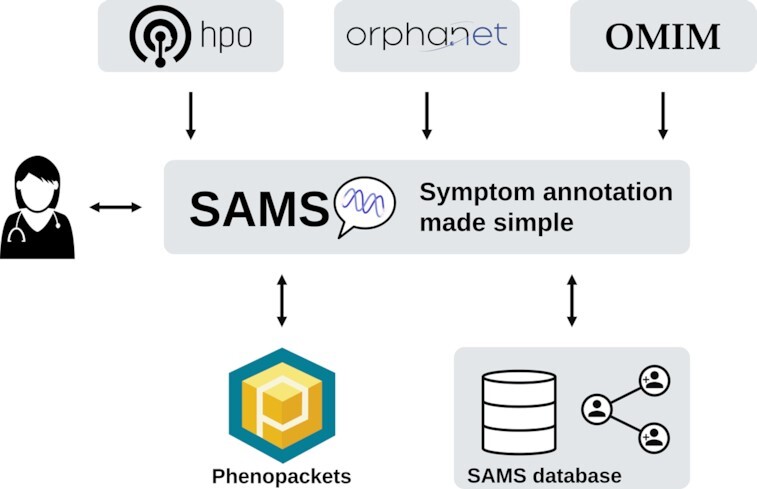
Deep phenotyping with SAMS. Data can be saved in our database, shared with others and exchanged as Phenopackets.

## INTRODUCTION

Many clinical information systems store diagnoses but not the underlying clinical signs. This leads to a dramatic loss of information and hampers precision medicine. Patients suffering from the same disease—or labelled with the same ‘billing diagnosis’—may present very different clinical signs whilst patients with similar signs may have completely different diagnoses. When it comes to revealing the aetiology of diseases, a thorough description of the phenotype is indispensable. The same is also essential for a personalised treatment.

The analysis of phenotypes plays a key role in clinical practice and medical research, and yet phenotypic descriptions in clinical notes and medical publications are often imprecise. Deep phenotyping can be defined as the precise and comprehensive analysis of phenotypic abnormalities in which the individual components of the phenotype are observed and described (1).

For a long time, OMIM (2) has been the primary resource for Mendelian diseases in humans. However, whilst OMIM descriptions of a disease contain a list of clinical signs, it remains unclear which signs an individual patient suffering from the disease presents. The same problem arises with annotations from Orphanet (3) which includes non-genetic rare diseases as well.

This problem was addressed by the Human Phenotype Ontology (HPO) (4), which offers a hierarchically structured list of >10 000 clinical signs, along with their definitions and synonyms. In the last decade, the HPO has emerged into the standard way of annotating patients suffering from single gene disorders. However, the HPO is not limited to single gene disorders or rare diseases but also used to characterise complex (‘common’) diseases (5).

The HPO differs from other available clinical terminologies in several ways. First, the HPO has substantially deeper and broader coverage of phenotypes than any other clinical terminology (6), the HPO is not a simple terminology, but a full OWL (Web Ontology Language) ontology and thus a computational resource that allows sophisticated analyses, including logical inference (7). Finally, the HPO-based computational disease models are now indispensable for all current phenotype-driven genomic diagnostics software (e.g. eXtasy (8), Exomiser (9) or MutationDistiller (10)).

To facilitate the exchange of phenotypic data, the Global Alliance for Genomics and Health (GA4GH) has recently suggested the Phenopacket schema. Phenopackets cover data for diagnosis and research of all types of disease including Mendelian and complex genetic diseases, cancer and infectious diseases (11). They are designed to be used across a comprehensive landscape of applications including biobanks, databases and registries, clinical information systems such as Electronic Health Records, genomic matchmaking, diagnostic laboratories and computational tools. A Phenopacket is a standard representation of an individual's medically relevant data, providing a computable case report of either a single medical encounter or a time course that can represent the entire medical history of an individual (12).

In the past, a major challenge to the use of the HPO in human genetics and other fields has been the lack of user-friendly and simple tools to browse the HPO hierarchy when phenotyping patients and to store their phenotype (i.e. present and explicitly absent signs) in a computer-readable fashion. With SAMS (‘Symptom Annotation Made Simple’), we aim to close this gap, providing both intuitive ontology browsing and search functions as well as a number of features intended to support translational research. SAMS supports the Phenopacket standard for data exchange.

SAMS offers four main modes:

1. Creation of a Phenopacket on the fly.
2. Use as a database to store and retrieve patients’ phenotypes.
3. Self-phenotyping of patients or their relatives and sharing their data with their doctors.
4. Integration into other applications.

## FEATURES

### Phenotyping a patient

**Figure 1.**
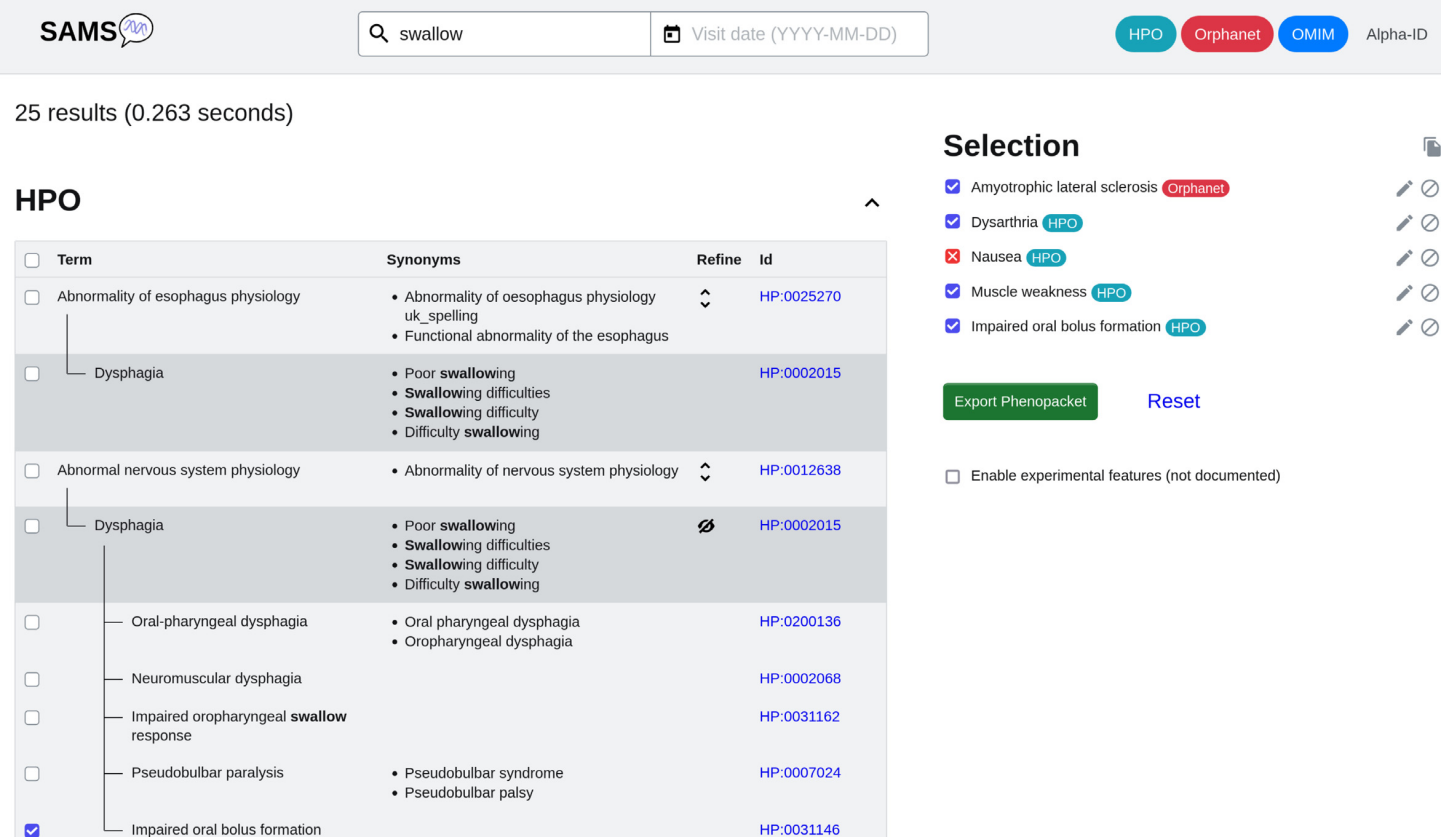
Phenotyping interface. Users can enter the signs or diseases they search for and suitable matches will be suggested by autocompletion, including HPO synonyms. These are the signs and diseases found for ‘swallow’ which lead to the term ‘Dysphagia’. The browse function available for HPO terms was used to find the more precise sign ‘Impaired oral bolus formation’. On the right, present and absent signs and diseases are shown. The complete record can either be saved in our database or exported as a Phenopacket.

The obvious main task of SAMS is the sign-based phenotyping of patients. As shown in Figure 1, our interface offers an autocompletion mode if text is entered to find matches from the HPO, OMIM and Orphanet. In case of the HPO, the autocompletion also includes synonyms. Selecting HPO terms allows users to browse the HPO tree to find closer matches, e.g. ‘Impaired oral bolus formation’ [HP:0031146] instead of ‘Dysphagia’ [HP:0002015].

Signs and diseases can be marked as present or absent (i.e. explicitly excluded by clinical examination).

The interface also offers a simple copy and paste function to directly insert a list of signs or to copy HPO IDs, e.g. to include them in a manuscript.

### Exporting a Phenopacket

Phenotypic data can either be stored in our database (see below) or exported as a Phenopacket for sharing or use in different applications. If patient data is stored in our database, all visits will be included in the Phenopacket hence providing a more comprehensive view over the course than data obtained in a single visit. This is especially important for non-monogenic disorders where the signs and symptoms may show many more changes over time than in congenital diseases.

### SAMS database

SAMS offers a light-weight database to enter, store, and retrieve patient signs, symptoms, and diagnoses. The database does not store any other data for each patient, except for their sex and consanguinity, the date of a visit, and a pseudonymised ID. The database does not allow storing names, birth dates or places. In the database mode, users are able to define as many patients as they wish, add an unlimited number of visits, study the course of diseases and symptoms over time, and export and import phenotypic data in the new Phenopackets format (12).

To protect patient data, a login is required to use our database and to share data within the database. All other functions are available without registration.

### Patient course

**Figure 2.**
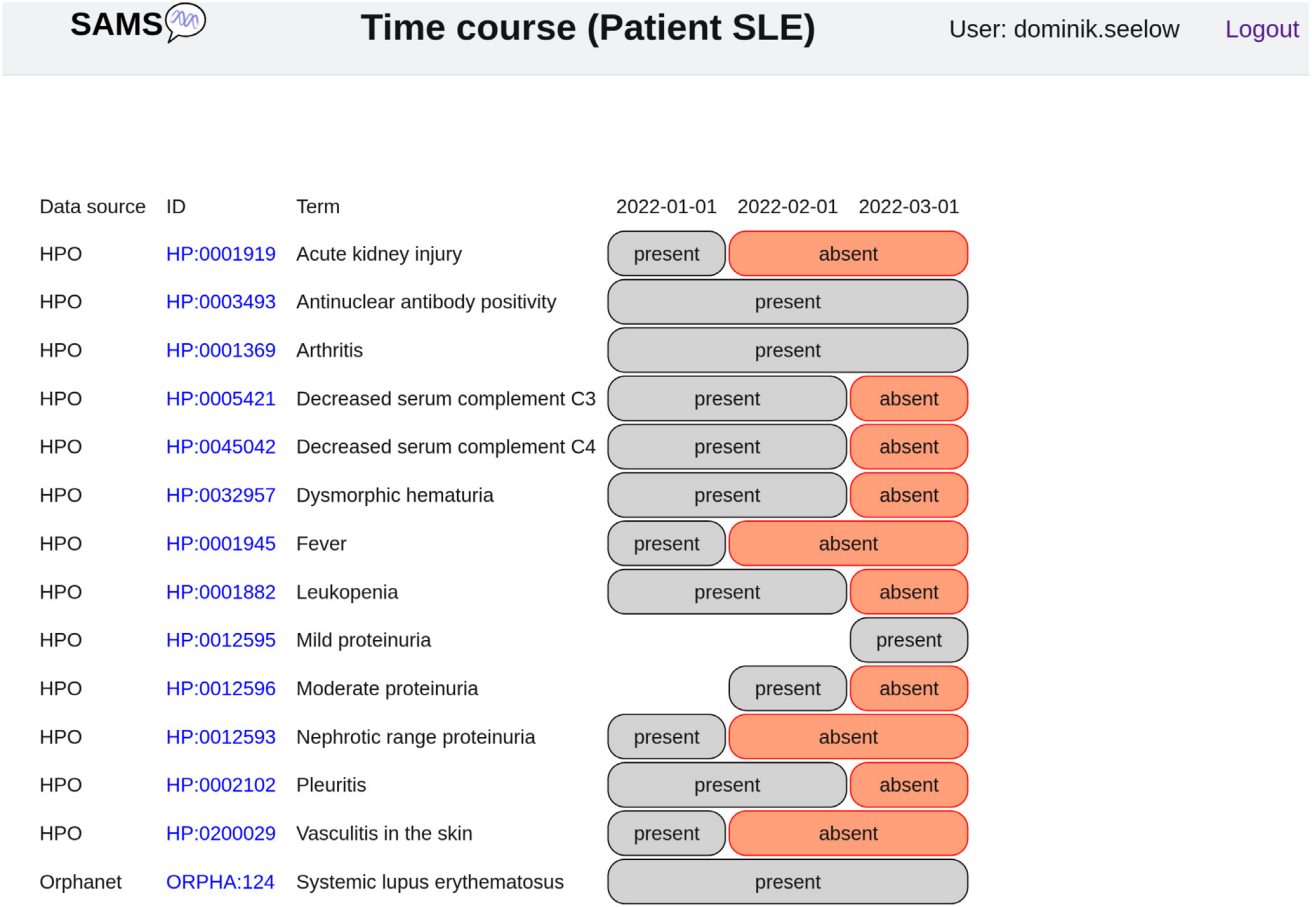
Time-course. This example shows the change of clinical signs in a patient suffering from *Systemic lupus erythematosus* under therapy. The diagnosis remains the same but many symptoms disappear.

An unlimited number of patient visits can be stored in the SAMS database. Whilst the diagnosis is unlikely to change in most cases, the signs and symptoms a patient presents may change over time or under treatment. SAMS provides a graphical view of the patient's course so that appearance or disappearance of clinical signs can easily be studied (Figure 2).

### Importing data

If the SAMS database is used, patient data can be imported from Phenopackets. All time points from the Phenopacket will be included as different visits.

Import of Phenopackets is limited to data usable from SAMS, e.g. signs and symptoms from the HPO and diseases or diagnoses from OMIM and Orphanet.

### SAMS for patients

Many patients (or their parents) suffering from rare diseases are keen on providing as much information about their disease as possible. SAMS allows patients to create their own account and phenotype themselves (or their children). These data can be shared with their doctors to give them regular updates about the course of the disease and the symptoms they encounter. This may lead to a better recording of symptoms or signs that are considered irrelevant by physicians and are therefore under-documented.

SAMS includes the layperson synonyms for HPO terms to facilitate the phenotyping process for non-clinicians.

### Sharing data

In addition to exporting phenotypic data as Phenopackets, users are free to share their records with other SAMS users. If they choose to share their data from within the database, they will receive a hyperlink which can be sent to their doctors or collaboration partners. Clicking the hyperlink will give access to the data. To prevent misuse, these links are only valid once and for 24 h.

It is not possible to edit shared data but by exporting and re-importing the patient profile, users can create their own dataset with full access permissions.

### Integration into other applications

The SAMS interface for entering phenotypes can be embedded into other applications, e.g. using an HTML5 iframe tag (https://www.genecascade.org/sams/iframe.html) without any registration. Phenotypic data can be exported as a Phenopacket. This allows the integration of SAMS-based deep phenotyping into local databases without using our database or transferring patient data beyond signs, symptoms, and diagnoses over the Internet.

Please note that some functions of the SAMS database (e.g. displaying the time-course of a patient's record) are not available using this mode.

## DISCUSSION

Thorough phenotyping is a key requisite not only for single gene disorders but also for other diseases: Whilst the ‘billing diagnosis’ will usually not change over time, clinical signs and symptoms may appear or disappear over time or under therapy. Retrieving them from written reports or even laboratory measurements is labour-intensive and error prone. Sign-based phenotypes enable automatic analyses of the patients, e.g. by finding sub cohorts of patients who had been labelled with the same ICD-10 diagnosis, or even the re-diagnosis on the basis of their symptoms. Another advantage is that the use of HPO terms allows a much faster collection of patients for studies such as clinical trials by searching for the signs they present or which are absent.

A suitable application of SAMS is to provide concise and precise information when patients are referred. Using international standards for the description of a patient's phenotype is less ambiguous than discharge letters may be and there is less need for translation if different languages are involved.

Since the database does not store any patient information other than their sex, consanguinity, visit dates, and the signs/diseases they present, sharing data either directly or as a Phenopacket does not reveal a patient's identity as sharing a discharge letter would. Embedding SAMS into remote applications reduces the transferred data to the visit date and the diseases and clinical signs. We also provide the source code of SAMS for on-site installation.

So far, SAMS is limited to the Human Phenotype Ontology, OMIM, and Orphanet but we are working on the integration of further data sources of phenotypic data. This does explicitly not include treatment data because we want to keep SAMS focussed and light-weight. With the possibility to use SAMS from within fully-fledged clinical information systems, a connection to further data sources and in-house medical records can be established.

We hope that a broader use of tools such as SAMS and the exchange of data using the Phenopacket schema will set new standards for deep phenotyping and foster research on and treatment of human diseases.

## OUTLOOK

We are currently working on the implementation of a guided differential diagnosis using the patients’ clinical signs but this is still in an experimental stage and therefore not available yet. For a better discrimination of relevant signs, we use the frequencies of HPO signs in Orphanet diseases (13).

For patients stored in our database, we will also implement the option to record worsening or improvement for symptoms that were present in the last visit.

We are also working on implementations of further annotation systems, with MONDO (14) being next.

Another upcoming feature is a granular setting for the permissions on shared data, i.e. whether or not users may add visits to shared data or modify the reports.

The software is already designed for using terms in languages other than English and we are working on a German version but this is still hampered by the state of the German translation of the HPO. Please contact us if you need other languages.

## DATA AVAILABILITY

SAMS is freely available at https://www.genecascade.org/SAMS/ and there is no login requirement if the database is not used.

The database schema and the source code are available for local installation at https://git-ext.charite.de/genecascade/sams.
